# Artificial Intelligence in Digital Pathology: What Is the Future? *Part 1: From the Digital Slide Onwards*

**DOI:** 10.3390/healthcare9070858

**Published:** 2021-07-07

**Authors:** Maria Rosaria Giovagnoli, Daniele Giansanti

**Affiliations:** 1 Faculty of Medicine and Psychology, Sapienza University, Piazzale Aldo Moro, 00185 Roma, Italy; mr.giovagnoli.univ.sap@hotmail.com; 2 Centre Tisp, Istituto Superiore di Sanità, 00161 Roma, Italy

**Keywords:** e-health, medical devices, m-health, digital-pathology, picture archive and communication system, artificial intelligence, cytology, histology

## Abstract

This commentary aims to address the field of *Artificial intelligence* (AI) in *Digital Pathology* (DP) both in terms of the global situation and research perspectives. It has four polarities. *First*, it revisits the evolutions of digital pathology with particular care to the two fields of the digital cytology and the digital histology. *Second*, it illustrates the main fields in the employment of AI in DP. *Third*, it looks at the future directions of the research challenges from both a clinical and technological point of view. *Fourth*, it discusses the transversal problems among these challenges and implications and introduces the immediate work to implement.

## 1. Introduction

Diagnostic pathology has undergone important changes and leaps forward by means of digitalization, which have allowed, from time to time, on the one hand, important changes in decision-making processes, and on the other, important changes in *workflow* and therefore in the *job description* of the insiders [1,2].

All this has had an important impact on the organization of work from one side and on the training of the figures involved in the activities on the other, having to prepare them to make the necessary changes to adapt them to the ever-changing *job description* and interactions with the tools (optics/mechatronics/informatics) in ever-more rapid obsolescence and gradually being more and more able to integrate with *eHealth* and *mHealth* [1,2,3,4,5,6].

We are moving from physical storage systems of slides to virtual storage of virtual-slides (i.e., *e-slides* or *digital-slides*) [3].

Old problems such as the organization of physical storage spaces are giving way to new problems such as physical (conservative) data security and cybersecurity.

Now there is less talk of archives and multi-archives for slides and more and more of how many petabytes or exabytes will be needed for the *e-slides*.

The changes have been so rapid that someone is starting to ask the fateful question:

Will the microscope still be needed as we know it today?

We can undoubtedly highlight how, to date, diagnostic pathology has gone through two important revolutions.

The great *innovations* in the field of the diagnostic pathology involved first the introduction of the immune-histo-chemistry in 1980 and second in the introduction of *next-generation sequencing for cancer diagnostics* around 2010.

The *first revolution* involved the introduction of digital pathology and therefore of the key elements from the *e-slide*, up to the acquisition system (video-camera or scanner) and to archiving system, the picture archive and communication system (PACS) for digital pathology [3].

This *second revolution*, if we leave out the era of robotic telepathology (which does not seem to have had a great impact in pathological diagnostics), had two important moments that we can call (a) the *revolution of digital pathology in eHealth* [5] with the possibility of accessing from the personal computer to PACS servers through virtual microscopy and (b) the *revolution of digital pathology in mHealth* [1] with the possibility of accessing the same servers from smartphones and tablets through a virtual microscope. As it has been highlighted by M Avanzo et al. in the review [6], nowadays, AI shows (1) the potentiality to access and correlate large amount of data and (2) direct prospective in the world of diagnostics.

Regarding (1), it is highlighted that today [6], both radiological and pathology images are stored in the PACS; moreover, with the introduction of electronic health records (EHRs), systematic collections of patient health information have been made available, which include both qualitative data, medical records, and laboratory and diagnostics information. AI, if applied to these big digital stores, could prove useful for epidemiological, clinical, and research studies.

Scientists in DP could benefit of AI from combining histopathological data obtained, analyzed, and shared with other sources of clinical data such as that obtained from omics and/or other databases with clinical data/demographic data and/or sources with BIG-DATA.

With regard to (2), it is highlighted that the development of the digital pathology [6] due to the introduction of whole-slide scanners and the progression of computer vision algorithms have significantly grown the usage of AI to perform tumor diagnosis, subtyping, grading, staging, and prognostic prediction. In the big-data era, the pathological diagnosis of the future could merge proteomics and genomics.

It is evident that AI is clearly helping to integrate information from multiple sources.

Furthermore, neural networks from AI are used, for example, to extract pertinent details from written notes from the slide representation.

In general, all of us are also expecting AI in DP as a deus ex machina to diminish the error rate and optimize the time of work.

## 2. Purpose

The contribution is in line with the Special Issue “The Artificial Intelligence in Digital Pathology and Digital Radiology: Where Are We?” https://www.mdpi.com/journal/healthcare/special_issues/AI_Digital_Pathology_Radiology (6 July 2021) [6].

The aim is to highlight, in light of the foregoing, the important aspects of the transitions towards DP and AI, highlighting: (a) the lights and shadows relating to the introduction of AI based on DP and (b) what could be the future directions to face to stabilize the AI in DP.

## 3. The Revolution of the Digital Slide

The introduction of *digital slides* (*e-slide* or *virtual-slide*) is undoubtedly a revolutionary change for the pathologist, comparable to that of the introduction of *google maps* for *cartography*.

Through digital pathology, it is in fact possible to navigate through the *e-slide* with reference to coordinates, perform *Zoom and Pan* operations and set references just as with *Google Maps*. Historically, DP in the first applications was faced with implementing telepathology connections [3]. In the first phases, there was talk of *telepathology* and not of DP. Conceptually, there were and still there are two methods to face *telepathology* (TP): *static TP* and *dynamic TP*. *Static TP* consists of the capture and digitalization of images selected by a pathologist or pathologist assistant, which are then transmitted remotely through electronic means. *Dynamic TP* consists of the direct communication between two different centers by using microscopes equipped with a *tele-robotic system* oriented to explore the slide, remotely operated by the *tele-pathologist* or an *assistant tele-pathologist* to reach a remote diagnosis. As an alternative solution between the two methods, widely increased, year after year, there is the *virtual microscopy* (*VM*) starting from the first applications. The latter does not refer to the *tele-control of microscopes*, whilst the glass is scanned as a whole, producing an *e-slide*, and a pathologist or the assistant pathologist can navigate remotely (via internet) inside this *e-slide* or *virtual slide* in a manner akin to a real microscope. It has to be considered that a single file representing the *e-slide* for pathology applications could reach several tens of gigabytes, more than in the case of applications of digital echography. Thus, the design of an appropriate visualization strategy is a basic core aspect.

Today, the diffusion of the VM was helped by: (a) the availability of fast internet connections; (b) the availability of consolidated visualization strategies; (c) the availability of power image acquisition cameras/scanners; (d) the availability of free visualization software.

We can clearly consider today that VM is an integral part of DP. Therefore, it can be used in biomedical laboratories with great potential. This can affect the organization of work and has the potential to change and improve training [2,4].

DP is not only *digital slides* [6]. However, it is impossible not to point out that *digital slides/e-slides* are a large part of DP.

For this reason, it is important to highlight some strategic aspects of this discipline of the VM and to consider how they evolved over the time.

### 3.1. The Difference between the Digital Cytology and Digital Histology

The cytologist and the histologist interact differently with the slides; therefore, when moving to the digital world, this aspect must be strongly considered. The cytologist analyses the cell while the histologist analyses the tissue. If we can make a comparison with architecture, the cytologist focuses on the brick and looks inside, whilst the histologist looks at the entire wall. For the cytologist to look inside the cell, it is particularly important to use the focus function, which is not needed by the histologist. This translates into cytology in an important need for digitization: that of allowing the focus function in the digital world. This is implemented with the creation of different digital layers to simulate fire through the *Z-stack* [3] function or other solutions that currently do not allow automatic implementation [7]. For these reasons, the *e-slide* in cytology requires an exorbitant memory occupation to cope with the *Z-stack*.

### 3.2. The Two Steps of the Revolution of the Digital Pathology: Integration into eHealth and mHealth

When we refer to the introduction of digital pathology, we must duly consider that there have been two important phases synchronized with the evolution of ICT that in healthcare have led to the developments of *eHealth* and *mHealth* applications.

Consequently, the first *client-server* informatic buildings had, in the era of *eHealth* developments, a strong component based on architectures based on PCs that connected via LAN/WAN.

**Figure 1:**
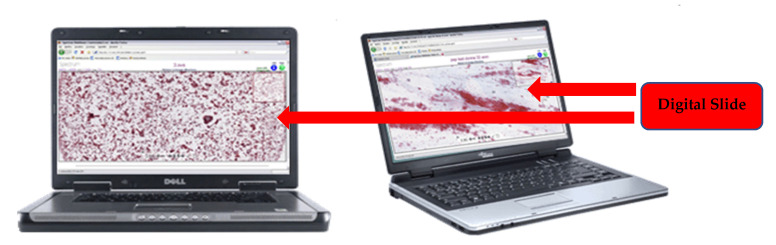
Access to the digital slides using eHealth.

Figure 1 shows an example of PC access to a *virtual slide* in the case of digital cytology.

Subsequently, starting with the release in 2008 of the first smartphones and/or tablets as we know them today [1], digital pathology has begun to find a fertile vehicle in *mHealth*.

**Figure 2:**
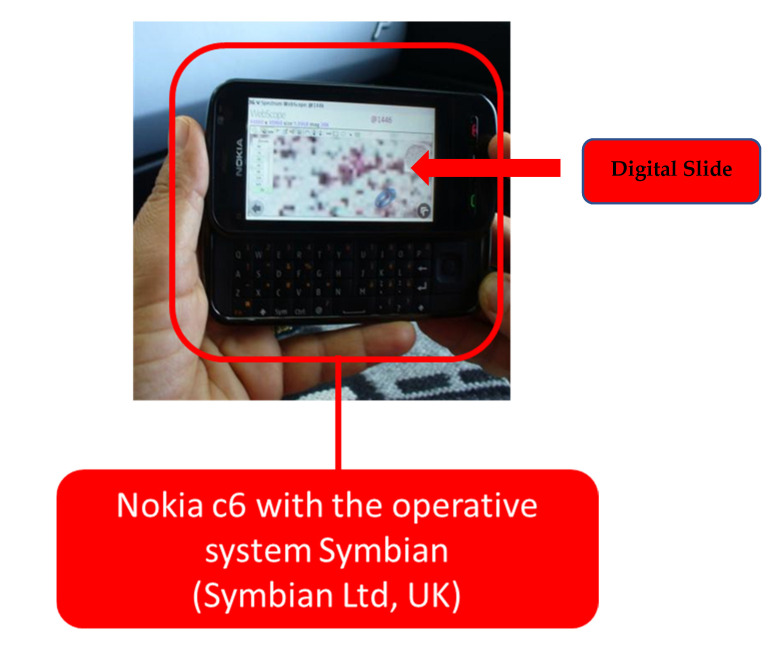
Access to the digital slides using mHealth not using the smartphone.

Figure 2 highlights a first application in *mHealth* in digital cytology with the Nokia c6 with the operative system Symbian (Symbian Ltd., Southwark UK) device, a border element between mobile phones and smartphones in a WI-FI hotspot.

**Figure 3:**
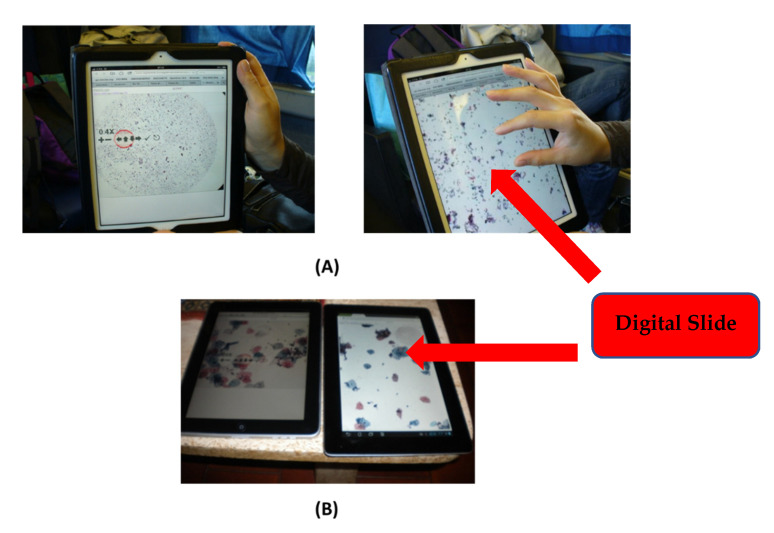
Access to the digital slides using mHealth (**A**) while navigating by a train without Wi-Fi; (**B**) a static connection.

Figure 3, again with reference to digital cytology, reports some accesses in *mHealth* by a tablet (A), from a train without WI-FI, and in other situations via smartphone (B).

### 3.3. The Acceptance of the Introduction: The HTA Studies Based on Properly Designed Surveys

A strategic aspect in the introduction of a technology is that of acceptance. Important aspects can be overlooked; moreover, problems of interaction with technologies that depend on generations could also arise. For example, the cytologist while navigating with the traditional microscope has a way of navigating and noticing important details with the side of the eyeball facing outward like that of primitive man to protect himself from attacks by ferocious predators. Switching to a PC-based method in *eHealth* first and *mHealth* on a smartphone or tablet later determines a radical change. Therefore, it is necessary to carry out targeted studies on the acceptance of technologies, focused on the actors, with reference to the most critical applications, as in the case of digital cytology. In the study reported in [5], we highlighted the importance of a health technology assessment approach based on a survey centred on the figures involved from a working point of view in digital cytology (which, as we have seen, presents major problems) in the *eHealth* phase. In the study reported in [1], we highlighted the importance of a health technology assessment approach with a similar configuration in the *mHealth* phase.

### 3.4. The Potentialities in the e-Learning/Remote Training

There is no one who does not see, in the COVID-19 era, that DP has important advantages in training regarding social distancing and the lightening of laboratories. Today, it is possible to access large databases and select targeted e-slide-based studies. Just to give an example, Leeds also has important archives with free access to the site https://www.virtualpathology.leeds.ac.uk/, accessed on 6 July 2021, [8].

See one of the many studies directly navigable with your browser in *eHealth* or *mHealth* by accessing the dedicated archive https://www.virtualpathology.leeds.ac.uk/slides/library/, accessed on 6 July 2021, [9], having fun with one of the many digital slides when navigating using a virtual microscope and simple mouse clicks https://www.virtualpathology.leeds.ac.uk/slides/library/view.php?path=%2FResearch_4%2FTeaching%2FEducation%2FManchester_FRCPath%2FDN%2F124388.svs, accessed on 6 July 2021, [10].

**Figure 4:**
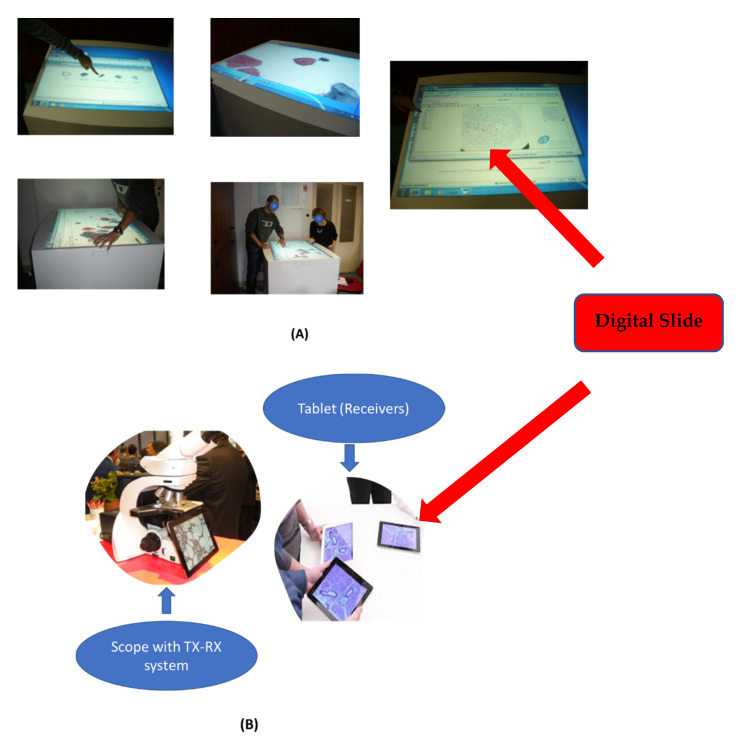
The two different types of training: (**A**) using a very large tablet; (**B**) using the DMSHARE.

In teaching, we highlighted the possibility of setting two important approaches [2]: (a) that of using very large tablets such as LIMS whiteboards or other ones in a finger-based and cooperative way to navigate virtual slides (Figure 4A), and (b) the other one based on a slide viewer or scope system with a webcam and a network transmitter to tablet/smartphone, even when not present (Figure 4B), such as, for example, the DMshare system (Leica Microsystems Co., Nussloch GmbH, Germany). Both have allowed to free up important resources in this pandemic period, such as dedicated laboratories. Of course, today, we can add a third dedicated method: one based on video conferencing with screen sharing.

### 3.5. The Standardization: A Slower Standardization Rate When Compared to Digital Radiology

The standardization of imaging in PD has had and is having a more tortuous road than digital radiology, wherein, thanks to DICOM, since the 1990s [11], a rapid process of digitization and compatibility of the diagnostic tools of the organs and functions has been initiated (echo, NMR, CT, PET, etc.).

Standardization in this area started with a slower process, and consequently, the compatibility between different manufacturers towards the standard has been delayed [6].

Today, DICOM WSI http://dicom.nema.org/Dicom/DICOMWSI/, accessed on 6 July 2021, [12] is used as standard in DP.

This standard considers the *whole slide images* (WSI)s in DP.

These images are exceptionally large.

As described in [12], a typical sample may be 20 mm × 15 mm in size and may be digitized with a resolution of 0.25 micrometers/pixel (conventionally described as *microns per pixel*, or *mpp*); here in the following we recall the characteristics reported in [12].

Most optical microscopes have an eyepiece which provides 10× magnification, so using a 40× objective lens results in 400× magnification.

Although instruments that digitize microscope slides do not use an eyepiece and may not use microscope objective lenses, by convention, images captured with a resolution of 0.25 mpp are referred to as 40×, images captured with a resolution of 0.5 mpp are referred to as 20X, etc.

The resulting image is therefore about 80,000 × 60,000 pixels, or 4.8 Gp.

Images are usually captured with 24-bit color, so the image data size is about 15GB.

This is a typical example, but larger images may be captured. Sample sizes up to 50 mm × 25 mm may be captured from conventional 1 × 3 slides, and even larger samples may exist on 2 × 3 slides.

Images may be digitized at resolutions higher than 0.25 mpp; some scanning instruments now support oil immersion lenses, which can magnify up to 100×, yielding 0.1 mpp resolution. Some operations described in [12] may further enlarge the data occupancy [12].

For example, a sample of 50 mm × 25 mm could be captured at 0.1 mpp with 10 Z-planes in the Z-stack, yielding a stack of 10 images of dimension 500,000 × 250,000 pixels. *Each plane would contain 125 Gp, or 375 GB of data, and the entire image dataset would contain a staggering 3.75 TB of data.*

## 4. Towards the Revolution of the Digital Pathology and Artificial Intelligence

### 4.1. What Is Emerging in the Application of the Artificial Intelligence in Digital Pathology

We carried out research with the aim of identifying the work to be completed, in terms of challenges and opportunities, towards stabilizing the use of artificial intelligence in DP, and then integrating what is highlighted with the considerations on digital pathology that we carried out in the previous section.

A quick look at PubMed with the following search key:

(*digital pathology* [Title]) AND (*artificial intelligence* [Title]) currently reports 17 works [6,13,14,15,16,17,18,19,20,21,22,23,24,25,26,27,28].

Among these works, one respects the search rule:

(digital pathology [Title]) AND (artificial intelligence [Title]) AND (COVID-19) [20]

that is, it relates to COVID-19.

What is highlighted by these works (many of which are editorial and/or opinion) from a general point of view are the following aspects. The first aspect is that when scholars talk about artificial intelligence in digital pathology, they refer more to the aspects of imaging and essentially histological imaging. The second aspect is that scholars begin to identify interesting perspectives—for example, in oncology [15,25] or in toxicology [14,24]. The last aspect, in line with our objective, is that scholars are interrogating the work to be completed in a prospective way [25,26,27,28].

Important perspectives have been identified for example:

- Through a review [15] on immuno-oncology.
- In a Special Issue [14] and in an opinion article [24] of a working group in pathological diagnostics in toxicology.
- Through a report [22] for the prediction of positive lymph nodes from primary tumors in bladder cancer.
- In cancer staging [18], it is well known that recent AI approaches have been applied to pathology images toward diagnostic, prognostic, and treatment prediction-related tasks in cancer. AI approaches according to this study [18] have the potential to overcome the limitations of conventional TNM staging and tumor grading approaches, providing a direct prognostic prediction of disease outcome independent of tumor stage and grade.

In the review that we have preselected as the only study linked to COVID-19 [20], it is highlighted that the effects of COVID-19 on research and clinical trials have also been significant with changes to protocols, suspensions of studies and the redeployment of resources to COVID-19 also useful for the applications of AI in DP. In this article, the authors explore the specific impact of COVID-19 on clinical and academic pathology and explore how digital pathology and artificial intelligence can play a key role in safeguarding clinical services and pathology-based research in the current climate and in the future.

We have identified *four prospective studies* that identify the critical issues and the work to be carried out [25,26,27,28].

*The first study*, although [25] it is not a review but an opinion, clearly identifies and discusses the critical issues in precision oncology by identifying some points on which to focus attention. The study aimed to provide a broad framework for incorporating AI and machine learning tools into clinical oncology, with an emphasis on biomarker development. *They discussed some of the challenges related to the use of AI, including the need for well-curated validation datasets, regulatory approval, and fair reimbursement strategies.*

*The second study* is an interesting review on the critical issues and the work still to be completed to arrive at the clinical routine [26]. This work highlights that while this is an exciting development that could discover novel predictive clinical information and potentially address international pathology workforce shortages, there is a clear need for a robust and evidence-based framework in which to develop these new tools in a collaborative manner that meets regulatory approval. *With these issues in mind, they have set out a roadmap to help academia, industry, and clinicians develop new software tools to the point of approved clinical use.*

The third study is an interesting review [27] that highlights that the advent of whole-slide imaging (WSI), the availability of faster networks, and cheaper storage solutions have made it easier for pathologists to manage digital slide images and share them for clinical use. In parallel, unprecedented advances in machine learning have enabled the synergy of artificial intelligence and digital pathology, which offers image-based diagnosis possibilities that were once limited only to radiology and cardiology. Integration of digital slides into the pathology workflow, advanced algorithms, and computer-aided diagnostic techniques extend the frontiers of the pathologist’s view beyond a microscopic slide and enable true utilization and integration of knowledge in new manner; therefore, it is important to focus on the WSI, now standardized in DICOM WSI and as radiologists and cardiologists move in line with the standards.

*The fourth study* is an interesting review [28] where the authors provide a realistic account of all the challenges of adopting AI algorithms in digital pathology from both engineering and pathology perspectives.

In the work, we found an interesting and shareable outline of the challenges of AI in digital pathology that naturally recalls what emerges in the other three interesting prospective studies [25,26,27,28] and lends itself well to the objectives of our study.

### 4.2. What Are the Perfectives and the Work to Be Carried out to Fully Integrate Artificial Intelligence in Digital Pathology?

#### 4.2.1. The Guiding Approach

In Section 3, we highlighted the characteristics and criticalities of the digital pathology on which the AI will have to rely and, in particular, which ones will have to be taken into account in routine applications.

We have, furthermore, seen above that to make AI a consolidated reality in digital pathology, it is necessary: (a) proceed with standardization processes including the need for well-curated validation datasets, regulatory approval and fair reimbursement strategies [25], (b) define roadmaps to help academia, industry, and clinicians to develop new software tools to the point of approved clinical use through concerted actions [26], (c) focus on the WSI, now standardized in DICOM WSI and, as the radiologists and cardiologists move in line with the DICOM standards [27], (d) provide a realistic account of all challenges of adopting AI algorithms in digital pathology from both engineering and pathology perspectives [28].

#### 4.2.2. Future Challenges

In their exhaustive review, Hamid Reza Tizhoosh and Liron Pantanowitz [28] recently categorized the challenges to be faced and also the evident opportunities. We fully share this useful approach organized as a useful grid. We summarize this briefly, referring to the review for an in-depth view.

##### Challenges in AI in Digital Pathology

**Figure 5:**
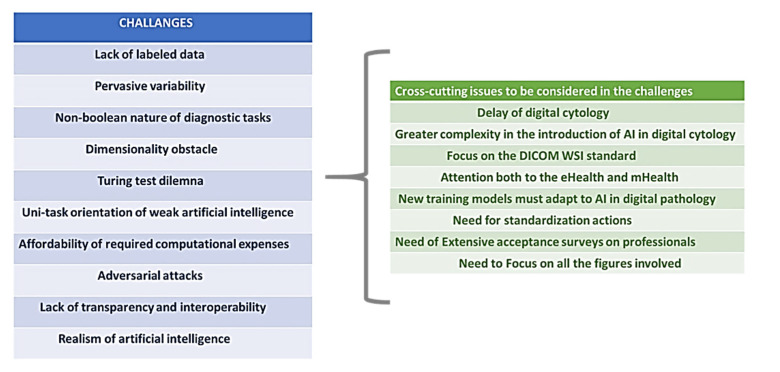
The challenges and cross-cutting issues emerging in the application of AI in DP.

The challenges that digital pathology presents for the integration of AI have been identified in [28]’s 10 *challenges* (Figure 5, table in the left)*:*

1. *Lack of labeled data*

The AI algorithms require a large set of good-quality training images. These training images must ideally be “labeled” (i.e., annotated). This is not easily feasible in DP.

2. *Pervasive variability*

There are several basic types of tissue (e.g., epithelium, connective tissue, nervous tissue, and muscle). The actual number of patterns derived from these tissues from a computational point of view is nearly infinite if the histopathology images are to be “understood” by computer algorithms.

3. *Non-Boolean nature of diagnostic tasks*

In pathology, not all can be summarized into two possible values such as “yes” or “no” (e.g., benign, or malignant). This is a too drastic a simplification of the complex nature of the diagnosis in this field. However, today, this is really not an issue; indeed, discrete variables (e.g., 1, 2, 3, 4) can be managed by machine learning, and there are also available methods based on regression machine learning for continuous variables, as reported in [29], for example.

4. *Dimensionality obstacle*

As we have highlighted in Section 3, the WSI deals with gigapixel digital images of extremely large dimensions up to 3.75 TB. Deep ANNs used in AI act on much smaller image dimensions (i.e., not larger than 350 by 350 pixels).

5. *Turing test dilemma*

The pathologist has the last word on the decision process when AI solutions are integrated in the workflow. Thus, full automation is probably neither possible, it seems, nor wise, as the Turing test postulates.

6. *Uni-task orientation of weak artificial intelligence*

What we consider today is mostly “weak AI” in contrast with strong AI, also called artificial general intelligence (AGI). Deep ANNs belong to the class of weak AI algorithms, as they are designed to perform only one task. Therefore, we need to separately train multiple AI solutions for different tasks. This obviously has implications.

7. *Affordability of required computational expenses*

Solutions with AI use graphical processing units (GPUs), highly specialized electronic circuits for fast processing of pixel-based data (i.e., digital images and graphics). These devices are expensive, and their adoption needs specific financial programs.

8. *Adversarial attacks—The noise in the deep decisions*

This is a common problem in AI; a little change in a pixel, for example, due to the noise may cause a completely different output in the ANN.

9. *Lack of transparency and interoperability*

The major drawbacks of artificial neural networks (ANN)s when used as classifiers is the lack of interoperability and transparency. Some consider ANNs to enclose a “black box” after the training.

10. *Realism of artificial intelligence*

There is currently optimism about the opportunities of ANNs, as has been highlighted above in the studies [13,14,15,16,17,18,19,20,21,22,23,24,25,26,27,28]. There are several difficulties with deploying AI tools in practice depending on the expectance and the objectives of the pathologist. There is no doubt that three are the preliminary requirements to improve this: (1) ease of use, (2) financial return on investment connected to the application, and (3) trust (such as, for example, the accountable performances).

##### Further Cross-Cutting Issues

We agree with the categorization identified by Hamid Reza Tizhoosh and Liron Pantanowitz [28], and I believe that it can be used as a reference for evaluating the future efforts of AI in digital pathology. Without introducing new challenges in detail, we would like to integrate the analysis with what emerged in Section 3 and in the other three selected prospective studies discussed above [25,26,27,28].

There are in fact aspects to be highlighted that act in a transversal way and are decisive for facing the 10 challenges identified in the categorization.

*Cross-cutting issues to be considered in the challenges* (Figure 5, table in the right).

N1. *Delay of digital cytology*. We have seen in Section 3 how digital pathology in digital imaging includes the two macro-sectors of digital histology and digital cytology. We have also seen above how in the studies addressed we refer mainly to the world of digital histology. This naturally translates into a foreseeable future delay of digital cytology due to less dedication on the part of scholars.
N2. *Greater complexity in the introduction of AI in digital cytology*. We highlighted in Section 3 that digital cytology needs the emulation of the focus function “to break through the sample”; this translates into the need to introduce the Z-stack, which can increase the WSI even 100 times compared to the histological case (up to 3.75 TB). This aspect must be duly considered.
N3. *Focus on the DICOM WSI standard*. As highlighted in [27], it is necessary to keep in mind the recent releases of standards to face large-scale studies on the introduction of AI in digital pathology and take inspiration from the world of digital radiology and cardiology, where the DICOM standards are now customary. This must apply to both digital histology and digital cytology. The weak AI mentioned above in *challenge 6* must navigate in extraction starting from standard WSI also to act on *challenge 10*, relating to concreteness and realism.
N4. *Attention both to eHealth and mHealth*. For AI, we need to consider both the worlds of *eHealth* and *mHealth*, where DP has stabilized through a path of acceptance [1,5].
N5. *New training models must adapt to AI in digital pathology*. Training models based on WSI and tablets and smartphones being remotely used must be able to include the provision of training also on *AI-based packages* and approaches. In this way, it is possible to integrate the two worlds of digital pathology and AI already in the training phase [2,4].
N6. *Need for standardization actions*. On the one hand, there is a need for manufacturers to adapt to standards [12]. On the other hand, as happens and/or is happening for telemedicine/tele-rehabilitation and alternative rehabilitation based on robotics, it is necessary to start a formal integration of digital pathology services connected with AI, as highlighted in [6]. This formal integration must have: a first step for consensus/acceptance paths between professionals that leads to important guidelines or recommendations. A second step that includes the provision of services in the healthcare offers the portfolio with coding of the service and reimbursement.
N7. *Need of extensive acceptance surveys on professionals*. This too is an important aspect interconnected with the previous ones. In Section 3, we highlighted how in the two phases of the introduction of digital pathology—*eHealth* and *mHealth*— there were important acceptance studies using HTA methods conducted on professionals through specific surveys [1,5]. These studies are also important in view of possible consensus conferences, or the activation of study groups dedicated to the activities of the previous points.
N8. *Need to focus on all the figures involved*. The introduction of AI in DP revolves various working figures in addition to the pathologist. These are the workers who will be involved in the reorganization of workflows, such as the clinical engineer and the biomedical laboratory technician [4,5]. These figures must be involved in standardization studies.

## 5. Conclusions and Work in Progress

### 5.1. The Evidences in the Study

In this study, the introduction of artificial intelligence in digital pathology was addressed. The study first tackled the second revolution in diagnostic pathology determined by the introduction of digital pathology techniques [1,2,3,4,5,6]. There is no doubt that most of the applications of AI take place in diagnostic imaging and that, therefore, AI rests on the imaging techniques used in digital pathology.

In analysing the important aspects of digital pathology, some important points/steps were noted:

- *The difference between digital cytology and digital histology.*
- *The two steps of the revolution of the digital pathology: integration into eHealth and mHealth.*
- *The acceptance of the introduction: the HTA studies based on designed surveys.*
- *The potentialities in the e-learning/remote training.*
- *The standardization: a slower standardization rate when compared to digital radiology.*

We then questioned the state of the next revolution that is anticipated due to the introduction of AI in DP. Through an overview of some important studies, some important development guidelines have been identified and, in line with the objectives of this study, the challenges to be addressed in detail and the transversal problems as they emerge both from the overview and from the characteristics and problems of digital pathology highlighted in the section dedicated to this discipline. The *10 challenges* were therefore recalled, starting from the grid identified in [28], and eight emerged transversal issues to be considered in these challenges were introduced and discussed (Figure 5).

### 5.2. Actual Developments and Future Work

All that is highlighted in the *cross-cutting issues* is, in a certain sense, of strong scientific interest and needs attention if we think of a routine introduction of AI in digital pathology. A point where we intend to contribute is that (no. 7) relating to acceptance based on surveys on key figures (no. 8), which is preparatory to standardization actions (no. 6 and no. 3–4). Inheriting the experience gained from previous studies [1,5], in which we had developed paper surveys for this purpose (relating to the introduction of digital pathology first in eHealth and then in mHealth), we are developing an electronic survey as a tool to be used with this purpose and we are using it to investigate this.

### 5.3. Limitations of the Study

The overview in the section was conducted with the search key “Artificial Intelligence”, wanting to stay on a higher and general level regarding the topic in line with the objectives of the study. Other more specific searches can be executed on aspects of a lower hierarchical level such as those relating to the algorithms of use. Artificial intelligence uses a myriad of different methodologies, techniques, and approaches that deserve specific review and research extended to non-medical databases, even if we are dealing with medical problems.

A long discussion deserves a targeted approach in the collection of *medical knowledge* in this area relating to supervised ANNs and unsupervised ANNs to collect successful and/or unsuccessful experiences.

A key search, for example, limited to the medical database PubMed of (digital pathology [Title]) AND (deep learning [Title]) led, at the date of this study, to 14 results [30], of which one was included in the one we made.

Another example of research on the same database of (digital pathology [Title]) AND (machine learning [Title]) led, at the date of this study, to seven results [31], of which four were included in the one we made above.

Such a research is more closely related to the specific performance of algorithms in DP and can highlight important development opportunities that must certainly be taken into account in any wide-ranging reviews.

## Data Availability

Not applicable.
